# Importance of selected genetic determinants on endurance performance and physical strength: a narrative review

**DOI:** 10.3389/fphys.2025.1568334

**Published:** 2025-06-26

**Authors:** Jiawei Yao, Falguni Saraf, Vishan Singh Rathore, Kinan Darkazanli, Yubo Liu, Mallikarjuna Korivi, L. V. K. S. Bhaskar

**Affiliations:** ^1^ Culture and Tourism College, Guangdong Vocational Academy of Art, Foshan, China; ^2^ Department of Zoology, Guru Ghasidas Vishwavidyalaya, Bilaspur, India; ^3^ Department of Physical Education, Guru Ghasidas Vishwavidyalaya, Bilaspur, India; ^4^ Institute of Chemical Engineering, Ural Federal University, Yekaterinburg, Russia; ^5^ Institute of Human Movement and Sports Engineering, College of Physical Education and Health Sciences, Zhejiang Normal University, Jinhua, China

**Keywords:** endurance, athletic performance, polymorphisms, angiotensin-converting enzyme, power, allele

## Abstract

Physical strength and endurance of an individual are vital for athletic performance, and minimizing the risk of injuries, especially during competitions. Other than training and diet, athletic performance is determined by genetic factors or heredity, which is less focused in sports science research. Genetic factors play a crucial role in greater cardiovascular endurance and muscular phenotypes, and thereby contribute to athletic success. Several genes and different polymorphisms are positively/negatively associated with athletic performance. This review delved into the intricate role of several genes and polymorphisms in different-population groups, and explored their impact on an individual’s ability to engage in athletic activities. Among several identified genes, the prominent genes, including *ACE*, *AGT*, *BDKRB2*, *NOS3*, *HIF1A*, *ACTN3*, *AMPD1*, *PPARGC1*, *SOD2*, *BDNF*, *VDR* and mtDNA are discussed in this study. These genes have been reported to play indispensable roles in endurance performance and power. Furthermore, genetic variations/polymorphisms within these genes are potential to impact various aspects of physiology, including cardiovascular function, muscle fiber composition, and metabolic efficiency. Genetic polymorphisms are recognized as contributing factors in determining the athletic capacity to engage and perform sustained physical activities in their respective sports. We emphasized the noteworthy discoveries from the existing literature, and precisely explored the association between particular gene polymorphisms and athletic prowess, with a specific focus on endurance-oriented sports (running, cycling, and swimming) and power sports. Understanding the genetic variations and their influence on endurance/power sports can offer valuable insights for athletes, coaches, and scientists in sports sciences, who strive to enhance athletic training strategies and performance outcomes in achieving success.

## Introduction

Endurance performance of an individual is influenced by several factors, and heritability is one of the important determinants. Genetic factors play a key role particularly in cardiovascular and muscular strength, and in the achievement of athletic success. Therefore, a favorable genetic profile is crucial for elite athletic performance and winning the competitions (Guth and Roth, 2013). According to research, the heritability values for performance-related characteristics to elite athletes are approximately 50% for optimum oxygen uptake (VO_2max_), 42%–46% for cardiac output, 40%–50% for muscle fiber type proportions and 67% for explosive muscle power (MacArthur and North, 2005). Therefore, it is advantageous to have a proper gene mix that is beneficial for athletes, particularly in terms of muscular strength and endurance. It is also said that favorable gene profile with appropriate training is advantageous for greater performance and athletic success (Guth and Roth, 2013). A recent meta-analysis identified a total of 50 genes and 94 different polymorphisms that are associated with various athletic characteristics, including endurance, strength, speed and power (Ferreira et al., 2024).

In addition to being a crucial component in many sports, endurance-related factors are also linked to excellent health and low mortality. The ability of an organism to perform a certain task for a prolonged period of time and remain active for a maximum period of time as well as its ability to resist, withstand and recover from, and have the immunity to battle trauma, wounds or fatigue is termed as endurance (Wan et al., 2017). Endurance usually comes into play in aerobic and resistance exercises. We should define each step to properly comprehend how the phases of exercises differ from one another. A group of long-lasting muscles may deliver sub-maximal force over an extended period of time or through repeated activities, the ability of the muscles to perform continuously without breaking down is necessary for endurance, and the metabolic system must be able to keep up with the removal of waste and supply of energy (Alghannam et al., 2021). These two systems should be “firm” in a way that allows them to continue operating for an extended period, but not always at a high level of intensity. Vitality is the ability of a specific group of muscles to generate their greatest amount of energy against an obstruction in a single motion. Energy is the ability of a group of muscles to produce their greatest amount of power in the least amount of time (Douglas et al., 2021).

There are mainly two types of physical endurances, such as cardiovascular endurance, and muscular endurance. Circulatory system has the potential for cardiovascular endurance, including the heart and lungs, to function for an extended period during activities like running, jogging, swimming, cycling, dancing and other similar sports. Heart and lungs work in tandem to supply the oxygen to muscles, make sure that an individual has everything that needed to complete the workouts. The Cooper Run (running as far as feasible in 12 min) is a popular test for determining the cardiovascular endurance (Cooper, 1968), however many trainers prefer the Step Test (stepping onto a platform for 5 min). These tests provide reliable assessments of cardiovascular endurance of an individual. The sports like football, hockey, and marathon running are popular in this category. The endurance of muscles is the ability to contract muscles for an extended period. For instance, during cycling, the leg muscles are exercised for minutes as compared to lifting or carrying something, in which muscles exercised just for a few seconds.

Exercise execution is a complicated attribute that influenced by a variety of contextual factors, including gender, social standing, training, and diet. However, innate traits, including genetics, also significantly influence the likelihood of developing into a human with high physical endurance (Eynon et al., 2013). This is because genes can influence muscular and cardiorespiratory functions, as well as responsiveness to the training stimuli, hence altering physical endurance (Rankinen et al., 2010). Athletic performance is mainly determined by the oxygen delivery, energy production, and recovery. These processes are tightly regulated by the genes involved in the metabolic efficiency, hypoxia response, cardiovascular regulation and muscle function. According to findings from the Heritage Study, the variance of key human endurance-related characteristics is likely 50% based on DNA genetic variations, meaning that the other 50% dependents on environmental variables, such as endurance exercise and diet (Simoneau and Bouchard, 1995). Genome-wide association studies (GWAS) have identified several genetic markers and polymorphisms that are associated with athletic performance traits, such as endurance, power, and strength. However, the direct physiological mechanisms by which these genes/polymorphisms influence athletic performance remain incompletely understood (Al-Khelaifi et al., 2019; Bıçakçı et al., 2024; Wang et al., 2022). An updated review stated that about 66% of variance in athletic status is associated with genetic factors, and the remaining variance is associated with other environmental factors, including diet, regular training, ergogenic aids and availability of medical and social support (Semenova et al., 2023). Although different genetic polymorphisms are reported to influence the athletic performance, a recent meta-analysis emphasized the need of future studies to explore the influence of polymorphisms in elite athletes from different background and sports disciplines (Ferreira et al., 2024). The published reviews till date are mainly focused on any one of the above physiological phenomena with few genes or polymorphisms. Although some reviews addressed the role of important genes, many of these reviews limited to emphasize the influence of gene polymorphisms on both endurance and strength of athletes from different population groups. The present review is a comprehensive, an updated and focused on every aspect of above-mentioned physiological adaptations across different population groups from different sports disciplines.

## Genes in endurance, power and strength performance

**FIGURE 1:**
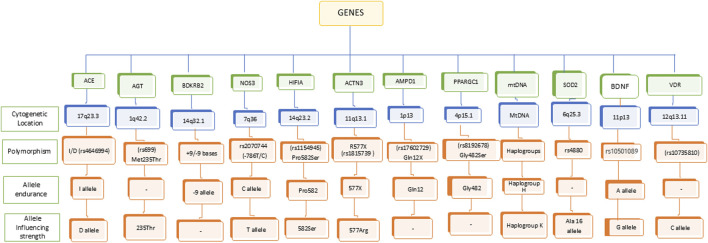
Genetic polymorphism associated with endurance and strength.

In the recent decades, several studies focused on attempting to understand the genetic influence on sports performance. Identification of key genetic variants that involved in endurance performance may help differentiate between elite and non-elite athletes. In this process, several gene variants have been identified to be associated with endurance performance and power-related performance (Semenova et al., 2023; Pickering and Kiely, 2017). The important genes involved in cardiovascular functions (*ACE*, *AGT*, *BDKRB2*, *NOS3*, *HIF1A*), muscle function and energy metabolism (*ACTN3*, *AMPD1*, *PPARGC1*, mtDNA), protection against oxidative stress (*SOD2*), neuro-muscular coordination (*BDNF*), and bone health (*VDR*) are known to determine the endurance and power in athletes (Figure 1; Table 1, 2). The other alleles, including *ACTN3* rs1815739C (Dogan et al., 2022; Bulgay et al., 2023), androgen receptor (*AR*) with ≥21 CAG repeats (Guilherme et al., 2021), *LRPPRC* rs10186876 A (Kikuchi et al., 2021), *MMS22L* rs9320823T (Semenova et al., 2023; Kikuchi et al., 2021), *PHACTR1* rs6905419 C (Semenova et al., 2023; Kikuchi et al., 2021), and *PPARG* rs1801282G alleles (Semenova et al., 2023) are involved for muscular strength. The most recent multiethnic GWAS conducted on world-class sprint and power athletes from West African and East Asian ancestry has uncovered a significant association of G-allele of rs10196189 in polypeptide N-acetylgalactosaminyltransferase 13 (*GALNT13*) with elite sprint and power perform compared to their geographically matched controls (Wang et al., 2025). Here we explored the influence of individual gene polymorphisms on athletic performance of different population groups.

**TABLE 1:** Genetic markers and their associations with athletic traits.

Genetic marker	Allele	Athletic trait	Association	References
*ACE*	I/D	Endurance	I/I associated with higher endurance performance	Myerson et al. (1999)
*AGT*	235 Thr	Blood pressure regulation	235 Thr/Th associated with higher blood pressure	Rankinen et al. (2000b)
*BDKRB2*	I/D	Blood flow regulation	Variations associated with endurance performance	Williams et al. (2004)
*NOS3*	G894T	Nitric oxide production	T/T associated with improved endurance performance	Saunders et al. (2006b)
*HIF1A*	Pro582Ser	Anaerobic glycolysis	Pro/Pro associated with sprinters, Ser/Ser with strength	Cieszczyk et al. (2011)
*ACTN3*	R577X	Muscle fiber composition	R/R associated with sprint performance, X/X with endurance	Pickering and Kiely (2017), Kim et al. (2014)
*AMPD1*	34C>T	Muscle energy metabolism	34C/C associated with elevated AMPD activity	Rubio et al. (2005)
*PPARGC1*	rs8192678 Gly482Ser	Endurance	Ser allele associated with endurance	Yvert et al. (2020)
mtDNA	Various haplogroups	Endurance and Power	Specific haplogroups linked to improved endurance or Power	Table 2
*SOD2*	Ala16Val	Oxidative stress	Val/Val associated with muscle injury	Ahmetov et al. (2014)
*BDNF*	rs10501089	Fast-twitch muscle fibers	A-allele is more in power athletes than in endurance athletes	Guilherme et al. (2022)
*VDR*	rs10735810	Bone mineral density	T/T associated with increased bone mineral density	Haussler et al. (2011)

**TABLE 2:** Association of mitochondrial DNA haplogroups with endurance and power in different population groups.

Haplogroup	Population	Relationship with endurance or power	References
Haplogroup L	Kenyan	Haplogroup L0 ↑, L3 ↓ in endurance athletes	Scott et al. (2009)
Haplogroup M	Korean	Haplogroup M ↑ in endurance athletes	Kim et al. (2012)
Haplogroup G	Japanese	Haplogroup G1 ↑ in endurance athletes	Mikami et al. (2011)
Haplogroup N	Korean	Haplogroup N9 ↑ in endurance athletes	Kim et al. (2012)
Haplogroup I	Finnish	Haplogroup I ↑ in endurance athletes and but not seen in sprinters	Niemi and Majamaa (2005)
Haplogroup H	Spanish	Haplogroup H ↑ higher VO_2max_ and higher mitochondrial damage during cycling exercise	Martínez-Redondo et al. (2010)
	Polish, Spanish and Finnish	Haplogroup H ↑ endurance than power	Maruszak et al. (2014), Castro et al. (2007), Martínez-Redondo et al. (2010)
Haplogroup V	Spanish	Haplogroup V ↑ in endurance athletes, but not in power athletes	Nogales-Gadea et al. (2011)
Haplogroup J	Spanish	Haplogroup J ↓ lower VO_2max_ and lesser stamina during cycling exercise	Martínez-Redondo et al. (2010)
	Finnish	Haplogroup J ↓ in endurance performance	Kiiskilä et al. (2021)
	Iranian	Haplogroup J ↑ in elite athletes	Arjmand et al. (2017)
	Finnish	Haplogroup J2 ↑ in sprinters but not seen in endurance athletes	Niemi and Majamaa (2005)
Haplogroup T	Spanish	Haplogroup T ↓ in endurance athletes	Castro et al. (2007)
Haplogroup F	Japanese and Korean	Haplogroup F ↑ in power athletes	Hwang et al. (2019), Mikami et al. (2011)
Haplogroup B	Korean	Haplogroup B ↓ in endurance athletes	Kim et al. (2012)
Haplogroup U	Iranian	Haplogroup U ↓ in elite athletes	Arjmand et al. (2017)
Haplogroup K	Finnish	Haplogroup K ↑ in sprinters but not seen in endurance athletes	Niemi and Majamaa (2005)
	Finnish and Polish	Haplogroup K ↓ in endurance performance	Maruszak et al. (2014), Kiiskilä et al. (2021)

## Angiotensin-converting enzyme (*ACE*)

The angiotensin converting enzyme (*ACE*) is the gene that most frequently investigated in relation to inherited physical endurance. This gene is responsible for encoding the angiotensin I-converting enzyme. The gene’s product is an essential part of the renin-angiotensin system (RAS), which regulates blood pressure, and plays a significant role in the overall efficacy of the body. One of the important genes associated with endurance performance is an insertion (I)/deletion (D) polymorphism (rs4340) in the *ACE* gene. Angiotensin I-converting enzyme activity in the blood is specifically associated with the insertion (I) rather than the deletion (D), which is associated with a higher level of endurance (Cieszczyk et al., 2009). Despite the *ACE* I allele being located within a non-coding intronic region, the insertion of an Alu sequence appears to impose transcriptional suppression, potentially via mechanisms, such as chromatin remodeling or interference with enhancer activity. This results in decreased *ACE* mRNA levels, leading to reduced enzymatic activity and decreased synthesis of angiotensin II, while concurrently maintaining bradykinin levels. The molecular mechanisms promote vasodilation, enhance skeletal muscle perfusion, and increase oxygen availability, thereby corroborating the noted correlations between the I allele and improved endurance performance. Nevertheless, the magnitude of this effect is relatively modest, and it’s influenced by additional factors such as training, environmental conditions, and polygenic interactions (Wu et al., 2013). A decrease in *ACE* activity results in a concomitant decrease in vasoconstriction, leading to an augmented supply of oxygenated blood to the actively contracting muscles (Sutar et al., 2024). Consequently, the existence of the I allele, which is related with reduced *ACE* activity, can be regarded as a favorable genetic mutation. Therefore, it is hypothesized that athletes who possess the I allele or have the II genotype may demonstrate a greater propensity for improved performance in endurance-focused athletic activities, such as running, cycling, and swimming. This is due to the crucial role of oxygen demand in these endeavors. The I allele and II genotype have been found to exhibit associations with divergent athletic performance characteristics, specifically in terms of speed/power versus endurance sports, as elucidated by a multitude of investigations involving elite athletes. The observed phenomenon elucidates a notable augmentation in the frequency of the I allele within a cohort of British athletes of Olympian caliber. Specifically, the aforementioned genetic variant exhibited an escalation from 35% among sprinters engaging in events exceeding 200 m, to a substantial 65% among distance runners participating in events surpassing 5,000 m (Myerson et al., 1999). It was observed that the short distance group of Russian athletes, including swimmers, skiers, triathletes, and track and field participants, exhibited a surplus of the D allele, accounting for approximately 72% of the observed alleles. Conversely, the middle distance group displayed an excess of the I allele, constituting approximately 73% of the observed alleles (Nazarov et al., 2001). According to the study conducted by Scanavini et al. (2002), a significantly lower percentage of anaerobic athletes in Italy, specifically 5.3%, possessed the II genotype, in contrast to the 33% of aerobic athletes. This finding was determined through the examination of VO_2max_ and Olympic ability, shedding light on the genetic variations between these two groups of athletes (Scanavini et al., 2002). The Finnish research conducted by Rankinen, et al., yielded congruent results, wherein athletes were categorized based on their maximal oxygen consumption levels. This categorization encompassed individuals engaged in cross-country skiing as well as running activities. The findings suggest a potential correlation between the allele and individuals engaged in endurance-based athletic activities (Rankinen et al., 2000a).

In terms of the distribution of *ACE* polymorphisms, it is noteworthy that the genotype frequency exhibited no considerable differences between the athletes and the controls. Specifically, among the female soccer athletes, the II genotype was observed in 40% of individuals, the ID genotype in 46.7% of individuals, and the DD genotype in 13.3% of individuals. Similarly, among the controls, the II genotype was present in 42% of individuals, the ID genotype in 48% of individuals, and the DD genotype in 10% of individuals. There was an absence of discernible disparity in the frequency of the I/D allele between the cohort of athletes and the group serving as a control (Wei, 2021). A study on the British Olympic-standard runners reported a positive association of *ACE* I allele with elite endurance performance. This was convinced by a greater I allele frequency among longer distance runners than controls (Myerson et al., 1999). The prevalence of the *ACE* DD genotype among young Columbian athletes engaged in strength-based athletic activities was observed to be approximately two-fold and 1.5-fold greater in comparison to those involved in endurance-based athletic activities and individuals from the control group, respectively. This observation implies that the *ACE* DD genotype exhibits a higher prevalence among individuals engaged in strength-based athletic activities compared to the remaining two cohorts (Ortiz et al., 2022). In contrast to this Iranian endurance athletes exhibited a higher frequency of the D allele (63.5%) compared to the control group (45.1%) (Shahmoradi et al., 2014). A latest study on Brazilian athletes revealed the higher frequency of *ACE* DD genotype in strength experts of elite group, and higher frequency of *ACE* ID genotype in strength expert sub-elite athletes. This study further emphasized that the DD homozygotes of the *ACE* belongs to elite group with strength phenotypes than the group of sub-elite and elite strength experts compared to elite endurance (de Albuquerque-Neto et al., 2024). Additionally, Brazilian football players possessing the DD genotype demonstrated with enhanced sprinting capabilities (Coelho et al., 2022). However, in Moroccan elite cyclists and field hockey players, *ACE* I/D is not associated with the risk of non-contact injury, suggesting that this genetic variant does not influence injury susceptibility in athletic population (El Ouali et al., 2025).

## Angiotensinogen (*AGT*)

The hepatic organ facilitates the synthesis of a vital protein known as angiotensinogen (*AGT*), which plays a crucial role in the renin-angiotensin aldosterone system. Upon the enzymatic action of renin, the substrate *AGT* undergoes cleavage by *ACE*, resulting in the formation of a distinct molecular entity known as angiotensin I (MacArthur and North, 2005). The angiotensin I does not exhibit biological activity in its current form. Nevertheless, angiotensin I has the potential to undergo subsequent enzymatic conversions, leading to the formation of angiotensin II (MacArthur and North, 2005; Crisan and Carr, 2000). This particular peptide plays a pivotal role in the regulation of blood vessel resistance and renal or sodium homeostasis in the human body, and thereby exerting a profound influence on the overall blood pressure dynamics (MacArthur and North, 2005). The elevated concentrations of *AGT* in the circulatory system induce an upsurge in the synthesis of angiotensin II, and thereby culminating the manifestation of hypertension (Da Eira, 2024). In a rodent experimental model, it was observed that the injection of AGT elicited a notable rise in the mean arterial blood pressure (Rodrigues et al., 2023). This increase was found to be directly proportional to the dosage of AGT administered, thereby establishing a dose-dependent relationship between the variables (Klett et al., 2001).

The AGT protein is derived through the process of protein synthesis, originating from the *AGT* gene. This particular gene is situated on chromosome 1q42.2, a specific region within the first chromosome (Shahid et al., 2022). The M235T (rs699) polymorphism in *AGT* is the most important gene of RAS, and associated with athletic status and performance. The *AGT* M235T polymorphism is characterized by a missense mutation, wherein a T to C substitution at nucleotide 704 leads to an amino acid alteration from methionine (M) to threonine (T) at the 235th position of the angiotensinogen protein (Makuc et al., 2017). This alteration does not directly influence the enzymatic function of the protein; however, it is associated with heightened transcriptional activity of *AGT* gene, resulting in increased plasma concentrations of angiotensinogen, which serves as the substrate for renin within the RAS. As a result, there is an augmented synthesis of angiotensin I, which is subsequently converted into its biologically active form, angiotensin II (Brasier et al., 1999). This compound serves as a powerful vasoconstrictor and plays a critical role in the regulation of sodium retention, maintenance of fluid homeostasis, and the process of vascular remodeling (Brasier et al., 1999; Fountain et al., 2025).

Individuals possessing the TT genotype (a homozygous for the threonine variant of *AGT* gene), consistently exhibit elevated expression levels of the *AGT* gene as well as increased circulating concentrations of angiotensinogen when compared to those with the MM genotype. The T allele is classified as a gain-of-function allele, which leads to an increase in RAS activity (Jeunemaitre et al., 1992; Puthucheary et al., 2011b; Bloem et al., 1997). This enhancement may improve cardiovascular efficiency, optimize oxygen delivery, and aid in the regulation of blood pressure elements that could contribute to power, strength, and potentially endurance performance. This phenomenon may also result in an increased left ventricular hypertrophy as a response to training, which can be beneficial for elite power athletes (Montgomery et al., 1998; Gomez-Gallego et al., 2009). The ‘heritage family study’ showed remarkable findings regarding the correlation between diastolic blood pressure and *AGT* Met235Thr polymorphism among middle-aged sedentary normotensive women. The findings concluded that the *AGT* M235T polymorphism is associated with body fatness, and the correlation between gene polymorphism and diastolic blood pressure is linked with fat mass in middle-aged sedentary normotensive women (Rankinen et al., 1999; Rankinen et al., 2000b). Genetic studies have suggested that increased *ACE* and angiotensin II serve as a skeletal muscle growth factors that further beneficial in improving the strength and power-related sports (Jones and Woods, 2003).

The TT genotype of M235T (rs699) polymorphism of the *ATG* gene reported to correlate with higher levels of angiotensin II, and increased blood pressure at rest as well as in response to intense exercise (Rankinen et al., 2000b). Previous investigation revealed that there were no notable disparities observed in the frequencies of *AGT* Met235Thr genotypes between Spanish elite athletes and the control group (Alvarez et al., 2000). A study conducted on Polish athletes revealed that the M235T (rs699) polymorphism in the *AGT* gene is associated with power but not associated with endurance performance (Zarebska et al., 2013). Another study on Polish Caucasian women has shown that the M235T genotype was associated with an improved single squat and average height of countermovement jumps, but no association was noticed for Wingate peak power and sprint running time (Aleksandra et al., 2016). Among 15 *AGT* polymorphisms, the AGTR2 C allele (rs11091046) carries of the angiotensin II, is reported to be associated with skeletal muscle development (increased proportion of slow-twitch muscle fibers), endurance athlete status and aerobic performance in Caucasian athletes (Mustafina et al., 2014). The C allele rs699 showed a good correlation to power performance, probably by the increased angiotensin II in resistance training male Caucasians (Ellis et al., 2017). A recent meta-analysis addressed the important role of gene polymorphisms in power athlete status, and highlighted that *AGT* rs699 Thr allele was significantly dominant in power athletes (Ipekoglu et al., 2023). Contrary, a latest meta-analysis stated that allele and genotype frequencies for *AGT* gene polymorphism were not significantly differ between control adults and endurance athletes (İpekoğlu et al., 2024).

It is important to note that both *ACE* and *AGT* genes are the part of the RAS linked with the cardiovascular function and muscle physiology. The individuals with the D allele of *ACE* ID polymorphisms are known to possess higher *ACE* activity that contribute to strength and power to perform high-intensity activities (Coelho et al., 2022; D et al., 2019). The M268T polymorphism of *AGT* gene involves a change of amino acid from methionine to threonine at position 268, and thereby accounts for 15%–40% of the variation in plasma angiotensinogen levels (Inoue et al., 1997). Carriers of Thr/Thr genotype (*AGT*) are reported to have higher levels of angiotensin II, and have an advantage in power and strength sports than that of individuals with other genotypes (Met/Met. Met/Thr) (Gomez-Gallego et al., 2009; Maciejewska-Skrendo et al., 2019). Hence the interaction between these polymorphisms may also determine the performance of an athlete. For example, the combined effect of *ACE* I/D and *AGT* Met268Thr polymorphisms significantly influences the modulation of endurance and strength phenotypes, primarily through their impact on the RAS, a critical regulator of blood pressure, fluid balance, and muscle perfusion (Alvarez et al., 2000; Puthucheary et al., 2011a). The carriers of insertion genotype (II) of *ACE* and Met/Met genotype of *AGT* gene may excel in endurance sports due to enhanced cardiovascular efficiency. In contrast, the carriers of *ACE* DD genotype and *AGT* genotype may show increased muscle performance and contractility, which is an advantage in strength and power-oriented sports (Gomez-Gallego et al., 2009). However, the joint contribution of *ACE* and *AGT* Met268 Thr polymorphisms remains counterseal due to the clear racial difference in the serum angiotensinogen level. This gene-gene interaction serves as a prime example of the polygenic characteristics inherent in athletic performance, underscoring the significance of integrated genetic profiles rather than relying solely on single-marker associations.

## Bradykinin 2 (*BDKRB2*)

Bradykinin 1 (*BDKRB1*) and the bradykinin 2 (*BDKRB2*) receptors are two distinct two-cell surface receptors via which bradykinin functions. The *BDKRB2* is also known as B2R, BK2, BK-2, BKR2 or BRB2. The *BDKRB2*, which has a strong affinity for kallidin (Lys-BK) and BK, is primarily responsible for mediating most physiological processes upon activation (Rex et al., 2022). The bradykinin 2 receptor or B2R is an essential G protein-coupled receptor (GPCR), which regulates the cardiovascular system as a vasodepressor. The bradykinin protein is involved in regulation of several key processes, such as cell proliferation, inflammation, smooth muscle contraction, glucose metabolism, oedema, pain, and modulation of vascular function (Rex et al., 2022; Shen and Zhang, 2023). The activation of *BDKRB2* causes greater skeletal muscle glucose uptake during exercise, increase blood flow in muscles, and thereby improve physical endurance performance (Williams et al., 2004). It has been documented that the human *BDKRB2* is consisted 359 amino acids and its molecular weight is 41 kDa (Prado et al., 2002). The single-copy gene that codes for *BDKRB2* is found on chromosome 14q32 and expressed in the majority of human tissues. The coding sequence is thought to be situated in exons 2 and 3 of the human *BDKRB2* gene, which is predicted to have a three-exon structure (Ma et al., 1994; Grenda et al., 2014).

The *BDKRB2* plays a significant role in muscle physiology. Genetic variations in *BDKRB2* have been associated with athletic performance. About the gene sequence, several investigations have found three polymorphisms in each exon and one in the promoter region. The insertion/deletion polymorphism (−9/+9, rs5810761) in exon 1 has been extensively studied in relation to genotypes and athletic status, as well as their association with cardiovascular diseases and hypertension (Fu et al., 2004; Saunders et al., 2006a). In contrast to the presence of a nine base pair (bp) repeat (+9), the absence of a nine base pair (bp) repeat (−9) is linked to heightened gene transcriptional activity, higher mRNA expression, and enhanced receptor activity in exon 1 of the *BDKRB2* gene (Lung et al., 1997). The −9 allele may thus be associated with improved skeletal muscle metabolic efficiency and higher endurance athletic performance (Williams et al., 2004; Brull et al., 2001). The *BDKRB2* receptor polymorphisms, −9 allele is associated with endurance phenotype in competitive swimmers (Zmijewski et al., 2016), while +9 allele is reported to be overexpressed in eastern European athletes. (Sawczuk et al., 2013). It is further stated that there was no association between *BDKRB2* -9/+9 polymorphism and athletic status in two cohorts of eastern European athletes (Sawczuk et al., 2013). The -9-9 genotype of rs5810761 which is rare, was found to be linked with increased skeletal muscle contraction efficiency in healthy individuals (Williams et al., 2004), and overexpressed levels were reported in Iron-man athletes (Saunders et al., 2006a). Latest study investigated the association *BDKRB2* variants with physical performance and muscle mass among older adults with low grip strength and low gait speed. The findings revealed that the rs5810761 -9-9 genotype was associated with lower arm fat mass, while the rs1799722TT genotype was associated with longer 6-minute walk distance and greater leg muscle mass among older adults (Shrestha et al., 2024).

## Nitric oxide synthase (*NOS3*)

Nitric oxide (NO) is identified as the most efficacious relaxation factor originating from the endothelium, characterized by its gaseous free radical nature (Félétou, 2011). The family of three enzymes, known as nitric oxide synthase (NOS) facilitates the conversion of arginine into nitric oxide. In comparison to neuronal nitric oxide synthase (nNOS, *NOS1*) and endothelial nitric oxide synthase (eNOS, *NOS3*), the inducible nitric oxide synthase (iNOS, *NOS2*) is often not constitutively expressed but can be induced in reaction to stress (Vecoli, 2014). An expanding corpus of research suggests that NO may partake in a multitude of physiological mechanisms that are pivotal for enhancing both aerobic and anaerobic efficacy. These mechanisms encompass glucose metabolism, specifically the uptake of glucose by human skeletal muscles during exercise. Additionally, NO is implicated in regulating the structure and function of skeletal muscles, facilitating the conversion of skeletal muscle fiber types, promoting mitochondrial ATP production, and influencing oxygen consumption within skeletal muscles (Gao, 2010). Furthermore, NO plays a vital role in the preservation, rejuvenation, and regulation of the myocardium’s oxygen utilization. Within the context of skeletal muscle, it is noteworthy to mention the identification of two distinct isoforms of NOS, namely, nNOS and eNOS. The predominant isoform identified in skeletal muscle is nNOS, whereas eNOS is primarily localized in endothelial cells and primarily functions in the regulation of vascular tone.

The *NOS3* gene, situated on chromosome 7 at the 7q36 locus, is responsible for the production of the endothelial NOS (referred to as *eNOS* or *NOS3*). The genetic sequence, spanning approximately 21 kilobases of genomic DNA, consists of 26 exons and is responsible for encoding a protein comprising 1203 amino acids in length (Marsden et al., 1993). Following an extensive examination of the *NOS3*, a multitude of polymorphic sites have been unearthed through a meticulous polymorphism screening process. The extensively researched and commonly observed genetic variations of the *NOS3* gene in exon 7 encompass the promoter-786T/C (rs2070744), G894T (also known as Glu298Asp or E298D or rs1799983), as well as the variable number tandem repeats (VNTR) and microsatellite (CA)n repeats located in intron 13, along with the 27 bp repeats situated in intron 4. The *NOS3* variants, specifically −786T/C and G894T, have been associated with various aspects of athletic performance. Research conducted by Saunders et al., on Caucasians, has demonstrated that G894T *NOS3* gene polymorphisms is associated with actual performance during the Ironman Triathlons, as well as the status of being an elite endurance athlete (Saunders et al., 2006b). Similarly, Gómez-Gallego and colleagues found a connection between these variants and power athlete status (Gómez-Gallego et al., 2009). The Glu/Glu genotype of *NOS3* is associated with greater lower limb strength and power, especially among elite soccer players occupying attacker and defender positions, indicating that the Glu298Glu may influence role-specific athletic performance (Petr et al., 2022). Furthermore, a study by Eynon et al. discovered a link between these variants and statuses of football player statuses (Eynon et al., 2012), as well as with the differentiation of elite power from endurance athletes (Gómez-Gallego et al., 2010).

Researchers have found that the *NOS3* −786T/C polymorphism in the promoter region of the *eNOS* gene influences its expression and NO production (Tanus-Santos et al., 2002; Oliveira-Paula et al., 2016). These changes in *eNOS* could help to preserve vascular function and supply oxygen during exercise (Tran et al., 2022). The T allele of this polymorphism links to increased eNOS activity due to increased interaction between transcription factors like activator protein 1 (AP-1) and nuclear factor-κB (NF-κB), and the promoter region (Oliveira-Paula et al., 2016; Maurer et al., 2020). This interaction boosts *eNOS* gene transcription. The increased expression of *eNOS* increases NO generation, a potent vasodilator that improves blood flow and oxygen delivery to skeletal muscles during exercise. The T allele’s effect on eNOS activity may improve endurance performance by improving vascular function and oxygen supply, especially during prolonged physical activity. However, this polymorphism’s effect on athletic performance is complex, and perhaps affected by other genetic and environmental factors, like training, food, and oxygen supply (Vostrikova et al., 2022). Physical prowess associated with the T allele of the *NOS3* c.-786T/C polymorphism (rs2070744) includes both power and endurance (Gómez-Gallego et al., 2009) because it improves the efficiency with which the cardiorespiratory systems work during exercise (Varillas et al., 2021). However, the polymorphism c.894G/T (rs1799983) T allele is a genetic associated risk for developing hypertension (Xin et al., 2009). In Spanish athletes, the −786T allele was found to be more prevalent (71%) among power athletes compared to endurance athletes (55%), with a statistical significance of P = 0.003. In a study, it was observed that elite football players possessing the −786C allele exhibited odds ratios varying from 1.879 to 4.032 when compared to other groups (Gómez-Gallego et al., 2009).

In a more precise manner, it is worth noting that there exist notable disparities in the occurrence rate of the *NOS3* 786T/C T variant, with percentages of 75.4% and 65.0% observed among Ukrainian athletes who emphasize endurance activities and the control group, respectively (Drozdovska et al., 2009). According to the study conducted by Gómez-Gallego et al., it was observed that both Spanish world-class endurance athletes and controls exhibited an equivalent frequency of the *NOS3* 786T/C T variant (Gómez-Gallego et al., 2009). Based on the findings presented by Drozdovska et al., it was observed that Ukrainian athletes with a power-oriented focus exhibited a significantly higher occurrence of the *NOS3* 786T/C T allele in comparison to the control group. The findings of these investigations were supported by two distinct studies, one involving elite Spanish athletes with a focus on power-oriented activities and non-athletic controls, and the other involving Italian power-oriented athletes (Drozdovska et al., 2013).

## Hypoxia-inducible factor 1α (*HIF1A*)

The process of glycolysis holds a crucial role as the primary source of energy in the absence of oxygen in humans. This metabolic pathway is regulated by a transcription factor known as ‘hypoxia-inducible factor 1α (HIF1α)’, which is encoded by a gene called *HIF1A* located on the 14q23.2 chromosome (Škrlec and Kolomeichuk, 2024). The HIF1α helps to regulate glycolysis in low-oxygen conditions (Basheeruddin and Qausain, 2024). Numerous genes involved in various physiological processes, including the metabolism of glucose, which includes glucose transporters and glycolysis-related enzymes, are controlled by HIF1α. In exon 12 of the *HIF1A* gene, there is a common genetic variation called Pro582Ser, which involves a change from a proline to a serine amino acid at position 582 due to a C/T change at bp 85 (also known as rs11549465) (Semenza, 2012). The T allele, which is less common, leads to an alteration that enhances HIF1α protein stability and transcriptional activity, consequently, may enhance glucose metabolism. The Ser allele (T allele) demonstrates a correlation with enhanced stability and transcriptional activity of the HIF-1α protein, even under normoxic conditions (Tanimoto et al., 2003; Keith et al., 2011). The increased activity diminishes protein degradation, facilitate accumulation of HIF-1α, which subsequently translocate to the nucleus to activate genes that play crucial roles in oxygen transport and energy metabolism, including angiogenesis and erythropoiesis, and several glycolytic enzymes (Tanimoto et al., 2003; Forsythe et al., 1996; Zimna and Kurpisz, 2015).

The aforementioned adaptations have the capacity to enhance capillary density, optimize oxygen delivery, and increase metabolic efficiency, thereby presenting potential benefits for endurance performance. Nonetheless, the impact of this polymorphism is complex and likely contingent upon various environmental factors, including altitude, training regimens, and nutritional intake (Tanimoto et al., 2003). Several studies investigated whether there was a difference in the distribution of the *HIF1A* Pro582Ser genotype between controls and Russian sprint/power-oriented athletes who rely heavily on anaerobic glycolysis for power performance. *HIF1A* Pro/Pro genotype were slightly more frequent in Caucasian male elite endurance athletes (Döring et al., 2010; Ahmetov et al., 2008). The 582Ser variant exhibited a higher prevalence among Russian strength athletes (Gabbasov et al., 2013). Weightlifters had a considerably greater frequency of the *HIF1A* 582Ser allele than the control adults. Furthermore, the frequency of the 582Ser allele was found to be increased with the level of achievement from sub-elite athletes to elite athletes, and to highly elite athletes (Ahmetov et al., 2008). A study conducted on Polish power-oriented athletes (weight lifter, short distance runners, and swimmers) revealed that the athletes had significantly higher frequency of the *HIF1A* 582Ser allele compared to sedentary controls (Cieszczyk et al., 2011). However, this difference was not observed in a group of Israeli sprinters (Eynon et al., 2010). In addition, the 582Ser allele was associated with an increased proportion of fast-twitch muscle fibers in the vastus lateralis muscle of all-round speed skaters (Ahmetov et al., 2015).

## Alpha-actinin-3 (*ACTN3*)

One of the most promising genes influence athletic performance is actin-alpha-3 (*ACTN3*), which is also known as “a gene for speed” (Pickering and Kiely, 2017). Fast-twitch (type II) skeletal muscle fibers encompass *ACTN3*, a protein belonging to the actin family that exhibits significant expression within muscular tissue. Through the orchestration of rapid-twitch muscle contractions, this particular protein possesses the capability to facilitate the cultivation of robust musculature when it is actively expressed within the glycolytic skeletal muscle, as elucidated by Yang et al. (Yang et al., 2003). The *ACTN3* gene, which codes for this protein, has been thrust into the forefront of genetic investigations as a result of the discovery of a nonsense polymorphism at position 577 (rs1815739), which has a major effect on the levels of *ACTN3* protein since the *ACTN3* gene contains an early stop codon (Bulgay et al., 2023). The most prevalent nucleotide at position 577, cytosine (C allele), codes for an arginine amino acid (arginine = R), with the alternative T allele coding a stop codon (stop = X). As a result, the CC genotype is known as RR and the TT genotype is known as XX in the scientific literature (referred to as R577X). Fascinatingly, it is found that over a billion people in the world are deficient in the homozygous absent XX genotype yet are believed to carry it (Eynon et al., 2013).

The ACTN3 R577X polymorphism stands out as one of the most extensively characterized genetic variants within the realm of sports genomics. The R allele, which facilitates the complete expression of α-actinin-3, enhances the performance of fast-twitch muscle fibers, thereby promoting activities that require power and sprinting capabilities. Notably, the RR genotype and R allele of *ACTN3* R577X are significantly more frequent in power athletes than in endurance athletes and non-athletes (El Ouali et al., 2024). In consensus to this, frequency of the RR genotype was shown to be significantly greater in Israeli sprinters (52%) compared to endurance athletes (18%) and controls (27.3%) (Eynon et al., 2009). In contrast, the X allele introduces a premature stop codon, leading to a total absence of α-actinin-3 and instigating a molecular transition towards an oxidative, endurance-oriented metabolic pathway. This encompasses improved mitochondrial functionality, modified calcium management, and fiber-type reconfiguration. Although there are notable correlations, particularly in power sports, the comprehensive impact of ACTN3 is influenced by polygenic factors, training regimens, and environmental conditions, rendering it a significant yet non-deterministic indicator of athletic capability (Pickering and Kiely, 2017). A transformation of fiber type towards fatigue resistance, as a remunerative mechanism for the absenteeism of functional *ACTN3* protein results due to an increase in the levels of alpha-actin two which was inferred from a study examining the effect of an *ACTN3* knock out in mice (Seto et al., 2013). Even athletes with a XX genotype exhibit this greater a tendency to engage in endurance activities similar to those shown in *in-vivo* investigations, as shown by the increased frequency of this particular allele.

The homozygous R allele of *ACTN3* and strength/power athletic traits currently have a strong relationship across different population groups (Cięs et al., 2011; Kim et al., 2014; Puthucheary et al., 2011b). Moreover, evidences indicate that there is a relation between a higher proportion of slow-twitch muscle fibers with the X allele genotype and elite endurance status (Ahmetov et al., 2011). This has been corroborated by a study in which the XX genotype frequency was higher among the Chinese female endurance athletes (21.2%) than that of the control group (15.8%) (Shang et al., 2010). In a group of Brazilian football players, it was discovered that those with the RR genotype outscored individuals with the RX and XX genotypes in short-distance sprints and jump tests (Pim et al., 2012). Another study on Brazilian swimmers and control adults reported that participants with XX genotypes of ACTN3 are more likely to belong to athlete group compared with that of control (de Albuquerque-Neto et al., 2024). The examination of the genotype distribution among individuals engaged in sprinting and distance athletics has yielded intriguing results. An analysis of these athletes has revealed that 44% have the RR genotype, 35% have the RX genotype, and 21% have the XX genotype. The associated p-value of 0.3143, which measures the statistical significance, indicates that there is no substantial correlation between *ACTN3* genotypes and the classification of individuals as either sprinters or distance athletes. This suggests that there is no noteworthy disparity in the distribution of genotypes when compared to individuals who do not engage in athletic activities (Dogan et al., 2022).

## Adenosine monophosphate deaminase (AMPD1)

Adenosine monophosphate deaminase (AMPD) is an enzyme that catalyzes the AMP to inosine monophosphate and liberate ammonia. The AMPD encoded by the gene *AMPD1* is important in the production of energy within the skeletal muscles, and regulate skeletal muscle energy metabolism during exercise. AMPD deficiency results in excessive accumulation of AMP during physical activity led to premature fatigue, muscle cramp and myalgia (Sabina et al., 1984; Fishbein et al., 1978). AMPD contributes to the modulation of muscle energy metabolism by altering the balance of the myokinase process in favor of ATP synthesis. The deficiency of AMPD isoform 1 (*AMPD1*) in skeletal muscle is a common genetic abnormality, with an allele mutation frequency of 12%–14% and complete loss of *AMPD1* in 2% of the general population (Gross et al., 2002; Morisaki et al., 1992). The human *AMPD1* gene (on chromosome 1p13) encodes the M isoform of myoadenylate deaminase, which is primarily generated at high levels in adult skeletal muscle. The common 34C>T (rs17602729) polymorphism in exon two of the *AMPD1* gene results in the formation of a premature stop codon (Gln12 X), culminating in a truncated and non-functional *AMPD1* enzyme (Leońska-Duniec et al., 2020). The absence of functional *AMPD1* significantly disrupts the purine nucleotide cycle, as it hinders the conversion of AMP to IMP, and this impairment results in the accumulation of AMP within skeletal muscle during periods of exercise. The accumulation of AMP serves to activate the AMPK (AMP-activated protein kinase) pathway, which is a crucial regulator of energy homeostasis. This activation facilitates mitochondrial biogenesis, enhances fatty acid oxidation, and increases glucose uptake—characteristics that are essential for aerobic metabolism (Hingst et al., 2020). Although this may hinder performance in high-intensity, anaerobic activities due to diminished ATP regeneration, it could potentially promote endurance-related adaptations in certain individuals. Nonetheless, the phenotypic manifestation of this polymorphism is shaped by interactions between genes, as well as between genes and environmental factors, rendering its effect on athletic performance intricate and contingent upon context. The skeletal muscle AMPD activity is incredibly low in individuals homozygous (XX) for the 34C>T (Gln12X) mutation of the *AMPD1* gene compared to the individuals with normal alleles (12Gln) (Fischer et al., 2007; Norman et al., 2008; Norman et al., 2001). The *AMPD1* 12X allele was observed in 4.3% of Spanish endurance athletes compared to 8.5% in the control group (Rubio et al., 2005). Whereas the *AMPD1* 12Gln allele is over represented (86.3%) in Lithuanian sprint/power-oriented athletes indicating that this allele may help to attain elite status (Ginevičienė et al., 2014).

When recovering from a 30-s workout, individuals with *AMPD1* deficiency exhibit a faster blood lactate formation and have less AMP deaminase activity. The AMPD-deficient group exhibits a more rapid strength drop throughout the 30-s Wingate cycling test, suggesting that *AMPD1* deficiency may have a negative impact on sprint or power performance (Fischer et al., 2007; Norman et al., 2008; Norman et al., 2001). Polish power-oriented athletes, including weightlifters, swimmers, and short-distance runners, showed a significantly lower frequency of the *AMPD1* 12X allele than controls participants (Cieszczyk et al., 2012). Similar results were observed in a sample of Russian power-oriented athletes (Fedotovskaya et al., 2013). A study on Lithuanian sprint and power athletes identified that *AMPD1* C allele may help to achieve elite status in sprint/power-oriented sports, while the T *AMPD1* allele is a unfavorable factor for athletics in sprint/power-oriented sports categories (Ginevičienė et al., 2014). In a recent study, Bulgay and colleagues showed that the *ADMPD1* G allele rs17602729 polymorphism may provide a beneficial effects to Turkish sprinters and power athletes (Bulgay et al., 2024). A most recent systematic review and meta-analysis demonstrated that the CC genotype of the *AMPD1* gene is significantly associated with elite status in both endurance and power athletes (Kartibou et al., 2025). This indicates that the individuals with *AMPD1* CC genotype may have a genetic advantage in sports demanding both aerobic and anaerobic capacity, due to improved muscle energy metabolism and fatigue resistance, regardless of specialization (Kartibou et al., 2025).

## Peroxisome proliferator-activated receptor gamma coactivator 1 (PPARGC1)

Peroxisome proliferator-activated receptor gamma coactivator 1 or PPARGC1 is a protein that regulates metabolic processes, and is encoded by the gene *PPARGC1A*. The genes, *PPARA* and *PPARGC1A*, coding PPARα and its coactivator PGC-1α, are highly expressed in skeletal muscle tissue that catabolizes fatty acids, and regulate remodeling of fiber type composition (Akhmetov et al., 2007; Pavlovic et al., 2023). It is also stated that higher endurance performance is achieved by the expression of these genes, which converts causes “fast-twitch” glycolytic type IIb fibers to “slow-twitch” oxidative type I/IIa fibers (Baoutina et al., 2007). The muscle fiber transition can happen even in the absence of exercise. This phenomenon in the absence of exercise can be attained by the expression of *PPARA*, *PPARGC1A* and *PPARD* genes that aids the transformation of type IIb to type I/IIa muscle fibers (Ahmetov et al., 2012). Previous research suggests that top-level endurance athletes may benefit from specific variants in *PPARA* and *PPARGC1A*, which influence gene expression and enhance metabolic efficiency (Hall et al., 2023; Eynon et al., 2013). These polymorphisms reported to be associated with improved mitochondrial biogenesis, fatty acid oxidation, muscle fiber-type conversion, glucose utilization, angiogenesis, and endurance performance, highlighting their potential role in athletic performance (Ahmetov et al., 2015).

The Gly482Ser (rs8192678) is the most common functional polymorphism is located in the exon 8 of *PPARGC1A* gene. Carriers of the 482Gly allele generally exhibit enhanced aerobic capacity, improved endurance, and a higher proportion of type I muscle fibers (Varillas-Delgado, 2024). The *PPARGC1A* Gly482Ser variant demonstrates a notable association with endurance, particularly through its role in mitochondrial biogenesis (Ahmetov et al., 2015). However, the existing literature indicate that the effect of Gly428Ser polymorphism vary depending on the sport to which they are applied. For example, it has been discovered that top athletes are less likely to carry the Ser allele in terms of power and endurance (Ginevičienė et al., 2011). However, other research indicates that the Ser allele is helpful for power-related tasks (Gineviciene et al., 2016). Therefore, the 482Gly allele was shown to be beneficial, but the Ser allele was claimed to discourage endurance exercises (Jin et al., 2016; Maciejewska et al., 2012). In consensus to this lower frequency of the Ser allele was observed in Israeli endurance athletes (25%) compared to the controls (43%) (Eynon et al., 2010). Interestingly, the Gly/Gly genotype and Gly allele were less frequent in elite Turkish track and field athletes than non-athletes (Tural et al., 2014). In a systematic review, G allele *PPARA* rs4253778 is said to be associated with endurance elite athlete status, and C allele *PPARA* rs4253778 is said to be associated with soccer (Petr et al., 2020). A comprehensive meta-analysis further substantiated the associations of Gly/Gly and Gly allele with enhanced endurance (Chen et al., 2019). A study examined the association between *PPARGC1A* rs8192678 A/A genotype and myosin heavy-chain isoforms (muscle fiber marker) among Japanese adults. The findings showed that *PPARGC1A* rs8192678 was significantly correlated with lower proportion of myosin heavy-chain-IIx and a higher proportion of myosin heaby-chain-1 in females (Yvert et al., 2020).

## Mitochondrial DNA (*mtDNA*) and mtDNA haplogroups

The majority of DNA is packed within the chromosomes located in the nucleus; however, it is noteworthy that mitochondria also possess their own distinct circular DNA known as ‘mitochondrial DNA’ (mtDNA). The human mtDNA, spanning a length of 16,569 base pairs, has a total of 22 transfer RNA genes, two ribosomal RNA genes, and 13 genes responsible for mitochondrial oxidative phosphorylation (Singh, 2023; Lim, 2024). These genes collectively play a crucial role in facilitating protein synthesis inside the mitochondria. On the other hand, individuals harboring *mtDNA* mutations typically represents with manifest symptoms, such as exercise intolerance, muscular weakness, and heightened lactic acid production (Andreu et al., 1999; Taylor and Turnbull, 2005). Evidence have shown that genetic factors location in nuclear genomes and mitochondria can influence the endurance performance of individuals. Studies further highlighted that *mtDNA* polymorphism influences the performance of general athletes, and *mtDNA* haplogroup appears to be associated with athletic performance of elite endurance athletes (Zanini et al., 2021; Maruszak et al., 2014). Further a lower frequency of heteroplasmy, and lower *mtDNA* copy number was documented in both power and endurance athletes of Polish origin (Piotrowska-Nowak et al., 2023).

Emerging evidence suggests that specific mtDNA polymorphisms, particularly within certain haplogroups, are associated with athletic performance of across diverse population groups. This association may be mediated by variations in mitochondrial efficiency and oxidative phosphorylation capacity (Table 2). For instance, haplogroup J in Caucasian associated with lower VO_2max_, efficiency of electron transport chain, and decreased production of ATP and reactive oxygen species (ROS), while the haplogroup H in Caucasians represented with higher VO_2max_ and greater physical stamina during exercise (Beiter et al., 2011). A study on elite Finnish endurance athletes has revealed the higher prevalence of *mtDNA* haplogroup H among the endurance athletes, whereas haplogroup K and J2 were not found among the endurance athletes. These two haplogroups are reported to be associated with longevity, make oxidative phosphorylation less efficient and might be disadvantage to endurance performance (Rygiel et al., 2016; Niemi and Majamaa, 2005). Another study on Spanish athletes reported that the mtDNA haplogroup T, specifically defined by 13368A was found to be significantly less frequent (negative association with athletic status) among elite endurance athletes (Castro et al., 2007).

Scott et al., compared the frequencies of *mtDNA* haplogroups among Kenyan national level athletes, international athletes, and general population. The findings revealed that the international athletes represented with higher proportion of L0 haplogroups and lower proportion of L3* haplogroups. These results imply that *mtDNA* haplogroups are the influential factors in elite Kenyan distance runner (Scott et al., 2009). In another study, male Spanish Caucasians with haplogroup J represented with lower VO_2max_ than that of individuals with non-J haplogroups. In this population, haplotype H claimed to be responsible for higher VO_2max_ and highest mitochondrial oxidative damage following incremental cycling exercise (Martínez-Redondo et al., 2010). A Spanish cohort study compared the frequency distribution of mtDNA haplogroups among the elite endurance athletes, power athletes, and a group of non-athletic controls. The findings disclosed a significant overexpression of haplogroup V in endurance athletes compared to controls, but not in power athletes (Nogales-Gadea et al., 2011). Another important finding from Finnish military conscripts revealed that the excellence in training or response to endurance performance was less among individuals with mtDNA haplogroups J or K compared to individuals with non-JK haplogroups (Kiiskilä et al., 2021).

Kim and colleagues performed a population-based study on Korean elite athletes, and reported that the distribution of *mtDNA* haplogroups M* and N9 were excess, whereas the haplogroup B was dearth in endurance/middle-power athletes compared with normal adults (Kim et al., 2012). Another study determined 20 mtDNA haplogroups in Korean population, and demonstrated a signification association of haplogroup F with athletic status (Hwang et al., 2019). Similar study on Japanese Olympic athletes revealed an excess proportion of haplogroup G1 in endurance/middle-power athletes, and greater proportion of haplogroup F in sprint/power athletes compared with control (Mikami et al., 2011). Top male Japanese endurance runners were found to have a significantly higher frequency of the m.5178C genotype of the m.5178CA polymorphism than the control adults. This m.5178C genotype in elite endurance runners may be beneficial for performance (Tamura et al., 2010). Another study observed that Japanese endurance athletes exhibit the variants m.152T>C and m.4343A>G, whereas power athletes display the variants m.151C>T and m.204T > C (Mikami et al., 2013). According to the study conducted by Maruszak and colleagues, Polish elite endurance athletes (Olympic/World class level) displayed a higher likelihood of belonging to mtDNA haplogroups H and HV, as well as possessing the *mtDNA* polymorphism m.16080G gene (favor to endurance), in comparison to both the control group and top power athletes (Maruszak et al., 2014). On the other hand, the Finnish endurance athletes do not possess haplogroups K and J2, whereas sprinters do exhibit these haplogroups (Niemi and Majamaa, 2005). A study assessed the association of mtDNA haplogroups with elite athlete status in Iranian population, and reported that haplogroup J was significantly over-represented, while haplogroup U was significantly under-represented in elite athletes (Arjmand et al., 2017).

## Superoxide dismutase (*SOD2*)

Superoxide dismutase (SOD) is the primary metalloenzyme in the antioxidant defense systems that quenches the superoxide anion radicals into oxygen and hydrogen peroxide. This reaction occurs in two steps, neutralizing the ROS while producing less harmful byproducts. Different forms of SODs localized in specific cellular components. The Cu, Zn-SOD or SOD1 is specific to the cytosol and mitochondrial intermembrane, while the Mn-SOD or SOD2 exists in the mitochondrial matrix and inner membrane (Fridovich, 1981; Rosa et al., 2021). Moderate exercise training has been shown to increase *SOD2* gene expression and decrease lipid peroxidation in untrained middle-aged men (Baghaiee et al., 2016). Several variations have been identified in the *SOD2* gene, including a non-synonymous variant that causes a change from alanine to valine in codon 16 of exon 2 (rs4880). It has been demonstrated that the 16Val allele this polymorphism is known to reduce the effectiveness of MnSOD in lowering oxidative stress (Shimoda-Matsubayashi et al., 1996).

Akimoto and colleagues reported that the *MnSOD* polymorphism (Val16Ala) may influence the release of muscle damage marker (creatine kinase) which might be a determining factor in performance among Japanese runners (Akimoto et al., 2010). A study conducted on Israeli endurance and power athletes demonstrated significantly higher frequency of 16Ala allele in athletes group compared to control group, however no difference found between power and endurance athletes. Furthermore, the frequency of Ala/Ala genotype was higher (29%) in international and Olympic-level athletes, while it was only 17% in national-level endurance and power athletes (Ben-Zaken et al., 2013). Another study on Russian and Polish athletes revealed that power/strength athletes were considerably less likely to have the *SOD2* Val/Val genotype than controls or athletes participating in low-intensity sports (Ahmetov et al., 2014). A recent study on Turkish elite athletes showed that the competitive endurance performance was in endurance athletes was significantly correlation with rs4880 polymorphism in the *SOD2* gene. However, no association was reported between performance and genotype frequencies within sprint/power athletes (Bulğay et al., 2023). Another recent study conducted on United Kingdom population showed that several single nucleotide polymorphisms (SNPs), including *SOD2* are positively associated with decreased body mass following an 8-week running program. The change in body weight was significantly associated with number of positive alleles present in exercised participants (Chung et al., 2024).

## Brain derived neurotrophic factor (*BDNF*)

Brain derived neurotrophic factor (BDNF) belongs to the neurotrophin family of growth factors that promotes neural plasticity linked to learning, memory, and recovery from brain injury (Numakawa and Kajihara, 2025; Cowansage et al., 2010). BDNF abundantly expressed in the hippocampus, cortex, and basal forebrain regions and supports the survival of existing neurons by the process of neurogenesis. Although, the expression and release of BDNF is determined by neuronal activity, the electrical activity stimulates *de novo* synthesis of BDNF leading to a significant increase in BDNF levels. Furthermore, high-intensity aerobic exercise and long-term training are known to elevate BDNF levels in humans and animals (Bathina and Das, 2015; Neeper et al., 1995; Pedersen et al., 2009). Low levels of BDNF have been attributed to a variety of psychopathological states, including Alzheimer’s disease, depression and schizophrenia. BDNF plays a crucial role in hypothalamic pathway that controls body weight and energy homeostasis, as well as in regulating the energy metabolism in peripheral organs (Pedersen et al., 2009). A recent GWAS on Russian population disclosed a dominant role of *BDNF* gene in the pathogenesis of alcohol dependency (Levchenko et al., 2022).

The human *BDNF* gene is composed of 11 exons and 9 promoters, spans 70 kb, and is located on chromosome 11p13-14. Several polymorphic variants have been described in the *BDNF* gene and some of them have been associated with activity-dependent *BDNF* expression. The Val66Met (rs6265) is a missense variant in exon 2, leads to substituting valine (Val) with methionine (Met) at codon 66 of the proBDNF protein. The Met allele carriers exhibit reduced *BDNF* secretion, thereby leading to impaired synaptic plasticity and neuronal survival (Shen et al., 2018). The study from Japan explored the influence of *BDNF* Val66Met polymorphism on athletic performance and psychological adaptation in swimmers and judo athletes. The findings highlighted that *BDNF* genetic variation may differentially influence the athletic performance and psychological adaptation across sport types (Asai et al., 2020). Patients who carry *BDNF* Val/Val genotype showed a greater reduction in posttraumatic stress disorder symptoms after exposure to therapy in combination with aerobic exercise, indicating that they benefited from exercise-augmented extinction learning (Bryant et al., 2024). The rs10501089 is another polymorphism that is located near the *BDNF* gene is linked to elevated levels of *BDNF* and fast-twitch muscle fibers in Russian power athletes. Furthermore, higher incidence of A-allele carriers was observed in power athletes compared with controls or endurance athletes (Guilherme et al., 2022). A recent study on Israeli females reported a significant association between *BDNF* rs925946T-allele carriers and obesity odds, which is affected by modifiable lifestyle factors, including physical activity, eating habits and sugar-sweetened beverages (Chermon and Birk, 2024). Martial arts athletes with *BDNF* G/G genotypes showed significantly higher conscientiousness scores compared to G/G genotype carriers in the control group (Humińska-Lisowska et al., 2022).

## Vitamin D receptor (*VDR*)

Vitamin D is a fat-soluble pro-hormone characterized by a complex metabolism and regulation. Vitamin D is located in intestinal cells, osteocytes, muscle cells, hematopoietic cells and the brain (Reijven and Soeters, 2020; Voltan et al., 2023). Along with its well-known role in calcium-phosphate metabolism and bone health, vitamin D also participate in a wide range of extra skeletal functions, including cell proliferation, antioxidant and immunomodulatory effect (Voltan et al., 2023). The functional ability of vitamin D is mediated by binding to its receptor (*VDR*), which located in many tissues, including skin, parathyroid glands, adipocytes, colon and small intestine, and able to bind hundreds of genomic loci (Reijven and Soeters, 2020; Sirajudeen et al., 2019). The presence of the *VDR* can also be observed in the cells of the human skeletal muscle. This receptor has the ability to interact with various metabolites of vitamin D, thereby exerting an influence on the metabolic processes occurring within the muscle cells (Pfeifer et al., 2002). Furthermore, the *VDR* also serves as a regulator in maintaining optimal calcium levels within the body by inhibiting the production of parathyroid hormone (Garfia et al., 2002; Haussler et al., 2011). In *VDR* knockout mice represented by decreased bone mass, hypophosphatemia, and increased calcitriol levels (Yoshizawa et al., 1997).

The human *VDR* gene is located on the long arm of chromosome 12, at position 13.11, exhibits an extensive repertoire of nearly 200 identified polymorphisms. *VDR* contains 6 promoter regions and eight exons two to 9. The DNA-binding domain (exons 2–4) interacts with the *VDR*E in target genes, whereas the ligand-binding domain (exons 6–9) binds 1,25(OH)D (Voltan et al., 2023; McCullough et al., 2009). Several health outcomes, such as the mineral content of bones, osteoporotic fractures, skeletal fractures, insulin resistance, muscular endurance, and susceptibility to various disorders like cardiovascular illness, osteoporosis, and sarcopenia, have been linked to genetic variations in the *VDR* gene (Kerr Whitfield et al., 2001; Banjabi et al., 2020). In individuals who possess the C allele, which is also referred to as the F allele, there is a notable distinction in the *VDR* protein compared to those who possess the T allele, also known as the f allele. This distinction arises from the rs10735810T/C transition that occurs specifically in exon two of the *VDR* gene, resulting in a shorter *VDR* protein (Cong et al., 2015; Meza-Meza et al., 2022).

The *VDR* genotypes of a cohort consisting of 206 individuals, both males and females aged 50–81 years, were studied to evaluate the *VDR* gene FokI and BsmI genotype in response to aerobic exercise and strength training. The findings revealed that a significant correlation between the *VDR* FokI genotype and femoral neck bone mineral density in response to resistance training, while no such association was observed in response to aerobic exercise (Rabon‐Stith et al., 2005). In various scientific investigations, the impact of the *VDR* rs10735810 T/C genotype on the overall bone mineral density of Japanese athletes was assessed. A study conducted on a group of 84 individuals who engage in weight-bearing, and a group of 48 individuals who participate in swimming. The findings demonstrated that the overall bone mineral density exhibited a greater degree of vulnerability to the effects of impact loading in individuals with the CC genotype. Athletes who exhibited the presence of the C allele demonstrated an augmented bone mineral density when compared with individuals who did not engage in athletic activities (Nakamura et al., 2002). Besides, Hopkinson et al., conducted a study using a sample of 107 persons diagnosed with severe pulmonary obstructive disease and 104 healthy individuals (Hopkinson et al., 2008). The study focused on the relationship between the FokI polymorphism in the *VDR* gene and quadriceps strength. The findings revealed that those with the CC genotype had reduced quadriceps strength compared to those with the TC or TT genotype (Hopkinson et al., 2008). Furthermore, other study conducted by Micheli et al. found significant variations in the frequencies of the DR FokI genotype among male football players at a medium-high level and sedentary controls (Micheli et al., 2011). The prevalence of the homozygous TT genotype of the *DR* gene was shown to be higher among young football players in comparison to a sedentary group with similar characteristics (Humińska-Lisowska, 2024). Additionally, a study revealed that 46 teenage soccer players from Brazil exhibited the FokI polymorphism, a genetic variation known to impact bone mass. TC genotype boys exhibited elevated whole body bone mineral content and density in contrast CC genotype boys (Tombari et al., 2024). The FokI polymorphism is believed to exert an influence on bone mineralization at various stages of bone growth, with particular emphasis on the first phases of maturation.

## Conclusion

Summarized evidence demonstrated that genetic factors substantially influence athletic performance, specifically in the realm of physical stamina. The heritability values about performance-related characteristics emphasize the significance of genetics. These characteristics include but are not limited to the optimum oxygen uptake, cardiac output, muscle fiber type composition, and explosive muscle power. It is important to note that these traits are subject to varying degrees of influence from genetic factors (Figure 1; Table 1). The complex interplay of genetic factors within individuals results in a wide array of outcomes, exerting profound influence on various qualities that are important for both elite athletes and overall wellbeing. The concept comprises two key physiological components (1) cardiovascular endurance, which dependent on circulatory system, (2) and muscular endurance, which pertains to sustained contractile performance of muscles. Genetic polymorphisms significantly influence these systems through multiple mechanisms, primarily by regulating the skeletal muscle fiber type distribution (slow-twitch and fast-twitch ratios), which directly affects the endurance or power performance of individuals. Genetic polymorphisms in key genes like *ACE*, *NOS3*, and *ACTN3* significantly impact physical endurance, strength/power, and athletic performance by influencing other factors, such as muscle fiber composition and blood flow. Specific alleles, including *BDKRB2* and *HIF1A* affect endurance and anaerobic capabilities, while variations in *SOD2* and *mtDNA* highlight the importance of mitochondrial function in athletics. Although genetic factors contribute to athletic potential, other factors like training, diet, and environment are also crucial. The growing understanding of these genotype-phenotype relationships enables precision and personalized training approaches but raises ethical issues around genetic testing and fair practices in sports.
